# Transaminases for the synthesis of enantiopure beta-amino acids

**DOI:** 10.1186/2191-0855-2-11

**Published:** 2012-01-31

**Authors:** Jens Rudat, Birgit R Brucher, Christoph Syldatk

**Affiliations:** 1 Institute of Process Engineering in Life Sciences, Section II: Technical Biology, Karlsruhe Institute of Technology (KIT), Engler-Bunte-Ring 1, 76131 Karlsruhe, Germany

**Keywords:** transaminase, beta-amino acid, high-throughput screening, biocatalysis

## Abstract

Optically pure β-amino acids constitute interesting building blocks for peptidomimetics and a great variety of pharmaceutically important compounds. Their efficient synthesis still poses a major challenge. Transaminases (also known as aminotransferases) possess a great potential for the synthesis of optically pure β-amino acids. These pyridoxal 5'-dependent enzymes catalyze the transfer of an amino group from a donor substrate to an acceptor, thus enabling the synthesis of a wide variety of chiral amines and amino acids. Transaminases can be applied either for the kinetic resolution of racemic compounds or the asymmetric synthesis starting from a prochiral substrate. This review gives an overview over microbial transaminases with activity towards β-amino acids and their substrate spectra. It also outlines current strategies for the screening of new biocatalysts. Particular emphasis is placed on activity assays which are applicable to high-throughput screening.

## Introduction

Since the discovery of transamination in biological systems ([Bibr B4][Bibr B42]) the significance of transaminases (TAs) for amino acid metabolism has been the subject of intensive research. Over the last 15 years, TAs have gained increasing attention in organic synthesis for the biocatalytic production of a wide variety of chiral amines and α-amino acids. This has been discussed in detail in a series of excellent reviews (Höhne and Bornscheuer 2009; Koszelewski et al. 2010; Taylor et al. 1998; Ward and Wohlgemuth 2010). Advantages in the use of TAs lie in mostly low-cost substrates, no necessity for external cofactor recycling and the enzymes' high enantioselectivity and reaction rate. For the synthesis of enantiopure β-amino acids only a limited number of TAs are available. Therefore efficient screening techniques for TAs with high activities as well as broader substrate specificity and different enantioselectivities are crucial for the successful application of transaminases for the synthesis of β-amino acids. Of particular interest are methods that can be used at small scale compatible with microtiter plates.

**Figure 1:**
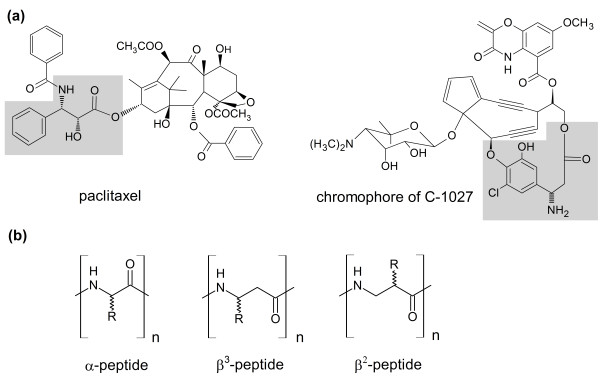
**(a) Examples of pharmaceutically important natural products containing a β-amino acid moiety: paclitaxel from the yew tree *Taxus brevifolia *and the chromophore of the chromoprotein C-1027 from the Actinobacteria *Streptomyces griseus***. The β-amino acid moieties are highlighted in grey. (b) Comparison of the backbones of α-, β^3^- and β^2^-peptides.

Enantiopure β-amino acids represent highly valuable building blocks for peptidomimetics and the synthesis of bioactive compounds. In order to distinguish positional isomers of β-amino acids, the terms β^2^-, β^3^- and β^2,3^-amino acids have been introduced by Seebach and coworkers (Hintermann and Seebach 1997; Seebach et al. 1997). With the exception of β-alanine and β-aminoisobutyric acid which constitute key intermediates in several metabolic pathways, β-amino acids are not as abundant in nature as α-amino acids. However, they occur as essential parts in a variety of biologically active compounds. Notable representatives are the antineoplastic agent paclitaxel (= Taxol™, Bristol-Myers Squibb) (Wani et al. 1971) and the chromophore of C-1027 (= lidamycin), a radiomimetic antitumor agent (Hu et al. 1988) (Figure 1a). β-Amino acids have drawn much attention as building blocks for synthetic peptides. They can form oligomers analogous to α-peptides with one additional carbon atom in the oligomer backbone (Figure 1b). These β-amino acid oligomers (= β-peptides) can form highly ordered secondary structures analogous to α-peptides (Iverson 1997; Koert 1997; Seebach et al. 1996; Seebach and Matthews 1997). β-Peptides are not recognized by most peptidases and thus not cleaved leading to a much higher *in vivo *stability compared to α-peptides (Frackenpohl et al. 2001; Gopi et al. 2003; Hintermann and Seebach 1997; Hook et al. 2004). It has also been observed that the substitution of only a few α-amino acids in a peptide by the corresponding β-amino acid lowers the proteolytic susceptibility (Horne et al. 2009; Steer et al. 2002). Apparently, the β-residues in mixed α/β-peptides tend to protect nearby amides from proteolytic cleavage. Interestingly, such mixed α/β-peptides often retain their biological activity (Aguilar et al. 2007; Horne et al. 2009; Montero et al. 2009; [Bibr B43][Bibr B54]

A plethora of chemical approaches have been established to produce chiral β-amino acids including (1) the resolution of racemic β-amino acids, (2) the use of naturally occurring chiral α-amino acids, and (3) asymmetric synthesis (Liu and Sibi 2002). As resolutions of racemic mixtures are complex and time-consuming procedures, the chiral pool of natural α-amino acids is limited and catalysts or chiral auxiliaries cause high costs, all of these strategies have their limitations when applied on an industrial scale (Weiner et al. 2010).

Several enzymes have successfully been tested to produce enantiopure β-amino acids from different starting compounds (for an overview see Liljeblad and Kanerva 2006). Most strategies resemble kinetic resolutions of *N*-acylated or esterified β-amino acids by hydrolytic enzymes, e.g. lipases (Tasnádi et al. 2008). Although industrially applied for certain products, this strategy is limited to a maximum yield of 50%, and so is the recently tested β-amino acid synthesis via Bayer-Villiger monooxygenases (Rehdorf et al. 2010). As the latter enzymes are cofactor (NADPH) dependent, these processes rely on cofactor recycling which is achieved by whole cell biotransformations, assumingly leading to side products as well as transport limitations depending on the substrate which moreover needs to be *N*-protected.

Two other novel approaches seem to be more promising as they - at least theoretically - can lead to a 100% conversion of the substrates used and thus overcome the inherent drawback of kinetic resolutions with the above described enzymes:

(1) Various aminomutases have been used for the conversion of aliphatic and aromatic α-amino acids to the corresponding β-isomers (for an overview see Wu et al. 2010a). Coupling the catalysis of a promiscuous alanine racemase with that of phenylalanine aminomutase (PAM) increased the production of enantiopure *(R)*-β-arylalanines from the corresponding racemic α-isomers (Cox et al. 2009). Using PAM in tandem with a phenylalanine ammonia lyase (PAL), various aromatic *(S)*-β-amino acids can be obtained (Wu et al. 2010b). These latter studies deal with one potential pitfall of utilizing these enzymes which lies in the reaction's equilibrium and the thus limited final yields of the desired products. Another limitation for application in industry is the usually low activity, leading to quite slow conversions. Moreover, many otherwise promising aminomutases require multiple expensive cofactors and strictly anaerobic conditions Wu et al. 2010a.

(2) A modification of the well established hydantoinase process is investigated for the production of enantiopure β-amino acids from dihydropyrimidine derivatives (Engel et al. 2011). The stereoselective hydrolysis of racemic phenyldihydrouracil to D- and L-*N*-carbamoyl-β-phenylalanine was shown which can be further hydrolyzed to the corresponding β-amino acid. However, at the moment this process lacks a suitable racemase (or alternatively an efficient chemical racemization) to gain a 100% yield.

In conclusion, even though several chemical and enzymatic routes (and chemo-enzymatic tandems) are applied and under intense investigation, there still is no gold standard for the preparation of enantiopure β-amino acids.

**Figure 2:**
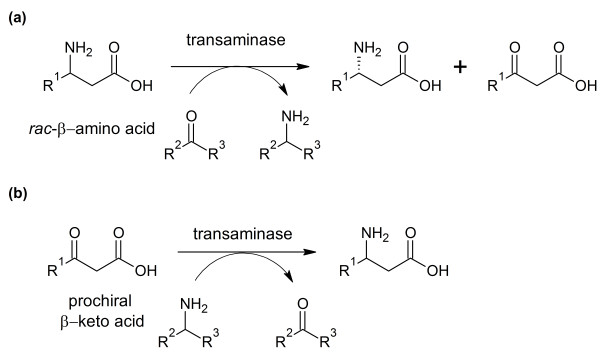
**Schematic reaction scheme of the synthesis of β-amino acids catalyzed by transaminases by (a) kinetic resolution of a racemic β-amino acid or (b) asymmetric synthesis starting from a prochiral β-keto acid**.

TAs can be applied either in the kinetic resolution of racemic β-amino acids (Figure 2a) or in asymmetric synthesis starting from the corresponding prochiral β-keto-substrate (Figure 2b). By asymmetric synthesis, a theoretical yield of 100% is possible. However, unlike α-keto acids, β-keto acids decarboxylate relatively easily under mild conditions in a mechanism involving a cyclic transition state (Bach and Canepa 1996). Therefore *in-situ *synthesis would be necessary. This can be achieved by enzymatic hydrolysis of the corresponding β-keto ester, as was already shown using a commercially available lipase from *Candida rugosa *(Kim et al. 2007) and a hog liver esterase (Banerjee et al. 2005).

### Reaction mechanism

Formally, the reaction catalyzed by TAs can be considered a redox reaction with the oxidative deamination of the donor in conjunction with the reductive amination of the acceptor. The reaction is divided into two half-reactions obeying a ping-pong bi-bi mechanism. TAs belong to the large and diverse group of pyridoxal phosphate (PLP)-dependent enzymes and are ubiquitous in living organisms playing an important role in amino acid metabolism ([Bibr B8][Bibr B9]). So far only the reaction mechanism of aspartate transaminase (EC 2.6.1.1) has been studied extensively, which is assumed to be typical of pyridoxal-5'-phosphate dependent transaminases ([Bibr B15][Bibr B56]). The reaction starts with the deamination of aspartate to α-ketoglutarate. In the resting enzyme PLP is covalently bound to the ε-amino group of a lysine (Lys258) in the active site of the apoenzyme forming the internal aldimine. Upon contact with the substrate, the bond between cofactor and apoenzyme dissolves, and PLP forms a Schiff base with the substrate (= the external aldimine). The free ε-amino group of Lys258 then acts as a catalyst for the 1,3-prototropic shift to form the ketimine. The ketimine is hydrolyzed to yield the keto acid and PMP. The following second half reaction consists of the formation of glutamate from α-ketoglutarate. Following the same reaction steps in reverse, the internal aldimine is regenerated ([Bibr B11][Bibr B18]).

### Classification of transaminases

Over the last decades several classification systems for TAs were established based either on function or evolutionary relationships. PLP-dependent enzymes are divided into seven major structural groups (fold types), which presumably represent five evolutionary lineages ([Bibr B17][Bibr B50]). Nonetheless, PLP-dependent enzymes encompass more than 140 distinct catalytic functions, thus representing a striking example of divergent evolution. This makes a correlation between sequence and function especially demanding. Recently, an extensive database has been built, which compiles information on PLP-dependent enzymes (Percudani and Peracchi 2009). Among the seven fold types of PLP-dependent enzymes, TAs occur in the fold types I and IV. Multiple sequence alignments by the Protein Family Database (Pfam) (Finn et al. 2010) led to the distinction of six subfamilies (classes) of TAs within the superfamily of PLP-dependent enzymes which are designated by Roman numerals (Table 1). The classes I and II, III and V all belong to the same folding type. Representatives of class I and II are aspartate TAs and aromatic TAs, of class III ω-TAs and of class V phosphoserine TAs. D-alanine TAs and branched chain amino acid TAs are set apart, pertaining to a different folding type, and unsurprisingly to a different subfamily. According to EC nomenclature, TAs are classified as transferases (EC 2) and not oxidoreductases, as the distinctive feature of the reaction is the transfer of the amino group. Names are generated according to the scheme *donor:acceptor transaminase*, e.g. asparagine:oxo-acid transaminase (EC 2.6.1.14). As of January 2012 81 different subgroups are listed under EC 2.6.1 (excluding deleted EC numbers). A broader classification based on the reaction catalyzed was introduced in the 1980s. TAs are divided into two groups: α-TAs which catalyze transamination of amino groups at the α-carbon and ω-TAs that act on the distal amino group of the substrate ([Bibr B6][Bibr B71]). According to this classification, all TAs acting on β-amino acids are considered as ω-TAs. It was observed that some ω-TAs are able to catalyze the transamination of primary amine compounds not bearing carboxyl groups (Yonaha et al. 1977). This led to an increasing interest in ω-TAs in recent years for the asymmetric synthesis of chiral amines of high enantiopurity ([Bibr B26][Bibr B37][Bibr B55]). Some biotechnologically important ω-TAs, such as the well characterized TA from *Vibrio fluvialis *JS17, have been denominated 'amine transaminases' accounting for their high activity towards amines while showing only low or no activity towards 'classical' ω-TA substrates, like β-alanine (Shin et al. 2003).

**Table 1:** Protein subfamilies of TAs according to Pfam; abbreviations: α-KG = α-ketoglutaric acid, PYR = pyruvate.

protein sub-families	Pfam ID	folding type	members	amino donor	amino acceptor	EC	α-/ω-TAs
I and II	00155	I	aspartate TA	L-aspartate	α-KG	2.6.1.1	α
		I	aromatic TA	L-phenylalanine	α-KG	2.6.1.57	α
III	00202	I	acetylornithine TA	acetylornithine	α-KG	2.6.1.11	ω
		I	ornithine TA	ornithine	α-KG	2.6.1.13	ω
		I	β-alanine:pyruvate TA	β-alanine	PYR	2.6.1.18	ω
		I	β-TA from *Mesorhizobium *sp. LUK	β-phenylalanine	α-KG or PYR	n.c.^1)^	ω
		I	4-aminobutyrate TA	4-aminobutyrate	α-KG	2.6.1.19	ω
IV	01063	IV	D-alanine TA	D-alanine	α-KG	2.6.1.21	α
		IV	branched-chain amino-acid TA	leucine	α-KG	2.6.1.42	α
V	00266	I	phosphoserine TA	phosphoserine	α-KG	2.6.1.52	Α
VI	01041	I	ArnB	UDP-4-amino-4-deoxy-beta-L-arabinose	α-KG	2.6.1.87	α

^1) ^n.c. = not classified

### Substrate spectra of TAs showing activity towards β-amino acids

#### Transaminases from wild-type microorganisms

Table 2 gives an overview over selected ω-TAs which show activity towards β-amino acids. β-Alanine:pyruvate TAs (E.C.2.6.1.18) and β-aminoisobutyrate:α-ketoglutarate TAs (E.C.2.6.1.22) are abundant in living cells, because they are involved in several important metabolic pathways such as pyrimidine degradation. Thus, numerous ω-TAs acting on aliphatic β-amino acids are known. Table 2 only includes a few examples which are of biotechnological relevance. In contrast, only few TAs with aromatic target compounds are known. TAs with high activity towards short-chain aliphatic β-amino acids such as β-alanine and β-amino-n-butyric acid often possess activity towards aromatic amines like α-methylbenzylamine, yet no or only low activity towards aromatic β-amino acids. The well-characterized ω-TA from *V. fluvialis *JS17 for instance possesses high activity to α-methylbenzylamine but also catalyzes the transamination of β-amino-n-butyric acid to the corresponding keto acid (Shin and Kim 2002). β-Alanine and β-phenylalanine do not serve as substrates Yun et al. (2004) reported an ω-TA from *Alcaligenes denitrificans *Y2k-2 which converts various aliphatic β-amino acids and amines but exhibits no activity towards β-phenylalanine. An exception is the ω-TA from *Caulobacter crescentus *which showed minor activity towards β-phenylalanine (Hwang et al. 2008). However, the relative activity of the wild-type enzyme towards α-methylbenzylamine was nearly 170-fold higher, towards β-alanine and β-amino-n-butyric acid even 300-fold 56. Shin and Kim (2002) constructed an active site model for the ω-TA from *V. fluvialis *based on its substrate spectrum. The authors tested a wide variety of donor and acceptor substrates and postulate a two-binding site model consisting of two pockets, one large and one small. The small pocket appears to accommodate no side group larger than ethyl groups and exhibits a strong repulsion for acidic groups. The carboxyl group is therefore always placed in the large pocket which results in the other side group of the substrate to be placed in the small pocket. Thus, high activities can be observed for β-amino acids with small side groups, e.g. β-amino-n-butyric acid, but not for large side chains like the aromatic ring of β-phenylalanine. 1-Phenylethylamine, on the other hand, does not possess a carboxylic group. Thus, the aromatic side-chain can be placed into the large pocket. For the confirmation of this model, the crystal structure will have to be elucidated. Jang et al. recently reported the crystallization and preliminary X-ray structure of the ω-TA from *V. fluvialis *(Jang et al. 2010). The crystal structure has not been released yet (Park and Jang 2010).

**Table 2:** Comparison of the substrate spectra of selected ω-TAs.

organism	amino donors				
	
	aliphatic β-amino acid			aromatic β-amino acid	aromatic amine
	β-ALA	β-ABA	γ-ABA	β-PHE	α-MBA
*Pseudomonas *sp. F-126 (Yonaha et al. 1976; Yonaha et al. 1977)	**++**	**++**	**+**		
*Moraxella lacunata *WZ34 (Chen et al. 2008)	**++**				
*Alcaligenes denitrificans *Y2k-2 (Yun et al. 2004)	**+**	**++**		**-**	**+**
*Caulobacter crescentus* (Hwang and Kim 2004)	**++**	**++**		**+**	**++**
*V. fluvialis *JS17 (Shin and Kim 2002)	**-**	**+**	**-**	**-**	**++**
*Chromobacterium violaceum* (Kaulmann et al. 2007)	**-**	**+**	**+**	**-**	**++**
*Arthrobacer *sp. KNK168 (Iwasaki et al. 2006)	**-**		**-**		**++**
*Alcaligenes eutrophus* (Banerjee et al. 2005)	**-**			**++**	
*Mesorhizobium *LUK sp. (Kim et al. 2007)		**++**		**++**	**+**
*Mesorhizobium loti *MAFF303099 (Kwon et al. 2010)				**++**	
*Variovorax paradoxus* (Banerjee et al. 2005)				**++**	
*Variovorax *sp. JH2 (Mano et al. 2006)				**++**	
*Variovorax *sp. BC114 (Brucher et al. 2010)				**++**	
*Burkholderia *sp. BS115 (Brucher et al. 2010)				**++**	

(++) high activity, (+) low activity, (-) no activity, () no data available; abbreviations: β-ALA = β-alanine, β-ABA = β-amino-n-butyric acid, γ-ABA = γ-aminobutyric acid, β-PHE = β-phenylalanine, α-MBA = α-methylbenzylamine.

Only a small number of TAs with high activity towards aromatic β-amino acids have been described (s. Table 2) and only two sequences, from the TA of the soil bacterium *Mesorhizobium *sp. LUK (GenBank: ABL74379.1) (Kim et al. 2007) and from the ω-TA *Ml0107 *from *M. loti *MAFF303099 (GenBank: NP_101976.1) (Kwon et al. 2010), have been elucidated and published to date. The TA from *Mesorhizobium *sp. LUK shows, as reported by the authors, the highest identity (53%) and similarity (66%) to a glutamate-1-semialdehyde 2,1-aminomutase of *Polaromonas *sp. strain JS666. Taking into consideration sequences which were submitted to Genbank since the publication of this article, the comparison of this amino acid sequence using blastp gives a putative aminotransferase class III from *Variovorax paradoxus *S110 (Gene ID: :7970445) as the closest match with 52% identities and 69% similarity. Interestingly, of the other transaminases reported to act on β-phenylalanine, one belongs to the species *V. paradoxus *(Banerjee et al. 2005) and two to the genus *Variovorax *([Bibr B5][Bibr B40]). The preliminary X-ray structure of the TA from *Mesorhizobium *sp. LUK has been published recently (Kim 2011).

Wild-type ω-TAs almost universally exhibit (*S*)-selectivity. Notable exceptions are the ω-TA of *Arthrobacter *sp. KNK168 ([Bibr B29][Bibr B30]) and its homolog, the commercially available ATA-117 (Codexis Inc.) as well as the TA from *Alcaligenes eutrophus *(Banerjee et al. 2005) 59. Svedendahl and coworkers (2010) could invert the enantioselectivity of an (*S*)-selective ω-TA from *Arthrobacter citreus *by single point mutation for their model substrate 4-fluorophenylacetone. This change in enantioselectivity was substrate-dependent. Whether or not this approach proves to be useful for the inversion of enantioselectivity of other TAs, remains to be seen.

#### Protein design of TAs for a modified or expanded substrate spectrum

Both rational design and directed evolution have been employed with the aim to enhance the activity of TAs towards aryl-β-amino acids 25. Hwang and coworkers (2004) reported the directed evolution of the ω-TA from *V. fluvialis *by error-prone PCR in order to increase activity towards β-phenylalanine. The best mutant exhibited a threefold activity increase in the conversion of β-phenylalanine compared to the wild-type. However, the yield of the transamination of β-phenylalanine was below 5% in 20 h. The same group later modified an ω-TA from *Caulobacter crescentus *which exhibited high activities towards short, aliphatic β-amino acids by site-directed mutagenesis (Hwang et al. 2008). A 3D model was constructed by homology modeling using a dialkylglycine decarboxylase as a template. This led to a threefold increase in activity for β-phenylalanine. Compared to the over 100-fold higher activity towards short, aliphatic β-amino acids, this is still quite low.

### Screening strategies for microbial TAs acting on β-amino acids

#### Enrichment culture and in-silico screening

The first directed screenings for microorganisms exhibiting TA activity towards β-amino acids were performed by enrichment culture using the desired β-amino acid as a major or the sole nitrogen source. Toyama et al. (Toyama et al. 1973) isolated the strain *Pseudomonas *sp. F-126 by enrichment culture from soil using a medium containing β-alanine. Further studies revealed it to possess an ω-amino acid:pyruvate TA with high activity towards β-alanine and other ω-amino acids (Yonaha et al. 1976). With a similar approach most of the currently known TAs with activity towards β-amino acids were discovered. As the sequence-function relationship among TAs is as of yet poorly understood, enrichment culture still constitutes the greatest source of new TAs active towards β-amino acids.

However, some attempts have been made to identify interesting TAs from the ever growing number of completely sequenced genomes. Kaulmann et al. (2007) used the sequence of the ω-TA from *V. fluvialis *for the *in silico *screening of novel TAs. They cloned and purified a putative TA from *Chromobacterium violaceum *which showed a similar substrate spectrum as the one from *V. fluvialis*. In a similar approach the previously described TA from *Caulobacter crescentus *was identified using the sequence of an ω-TA from *Alcaligenes denitrificans *as a template (Hwang et al. 2008). The novel TA exhibited high activities towards short, aliphatic β-amino acids and aromatic amines. Recently, Kwon et al. (Kwon et al. 2010) established the cell-free expression of computationally predicted putative ω-TAs, which circumvents cloning and expression procedures. As part of this study, the putative ω-TA *Ml0107 *from *M. loti *MAFF303099 was identified based on its sequence homology with the previously described TAs from *Caulobacter crescentus *and *V. fluvialis*. ω-TA *Ml0107 *exhibited activity to β-phenylalanine, 1-aminoindane and benzylamine.

The sequences of the two TAs with high activity towards aromatic β-amino acids (TA from *Mesorhizobium *sp. LUK (Kim et al. 2007), ω-TA *Ml0107 *from *M. loti *MAFF303099 (Kwon et al. 2010)) have, to the best of our knowledge, so far not been used for *in-silico *screening.

#### Activity assays for high-throughput screening

**Figure 3:**
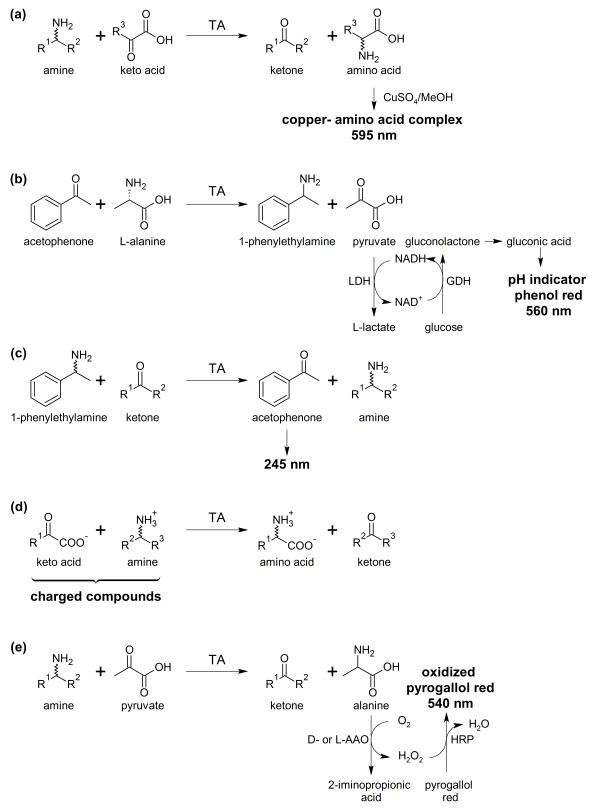
**Activity assays for the screening of novel transaminases (a) by formation of a blue copper complex with the produced α-amino acid, (b) by withdrawal of the produced pyruvate in a multi-enzymatic one-pot reaction system which ultimately leads to a pH drop, (c) by direct measurement of the absorbance of acetophenone produced from the transamination of α-methylbenzylamine, (d) by measuring the decrease in conductivity which results from the conversion of the two charged substrates to the uncharged/zwitterionic products and (e) by oxidation of the produced alanine which ultimately leads to the oxidation of the dye pyrogallol red by H_2_O_2 _in a multi-enzymatic one-pot reaction system**. Abbreviations: LDH = lactate dehydrogenase, GDH = glucose dehydrogenase, AAO = amino acid oxidase, HRP = horse radish peroxidase.

A major limiting step in the discovery, characterization, optimization and purification of new TAs lies in the determination of TA activity. This gave rise to the development of several high-throughput (HTP)-methods. Figure 3 gives an overview over HTP-assays which allow the screening for TA activity towards β-amino acids.

The first assay was realized by Hwang et al. (2004). It is based on the formation of a blue amino acid-copper complex by the α-amino acid produced in the TA reaction and a CuSO_4_/MeOH staining solution (see Figure 3a). The assay was tested using a great variety of aliphatic and aromatic β-amino acids as amino donors with good accuracy. Furthermore, by using both enantiomers of an amino donor separately, information on the enantiopreference of the studied enzyme could be gained. A disadvantage of this method consists in the fact that the staining solution inhibits the enzyme, so that it can only be applied as an end-point measurement. Additionally, this method does not allow the use of cell extracts as free α-amino acids disturb the reaction. Thus, enzyme purification is necessary. Therefore the application of this assay is rather limited. Coupling the determination of TA activity with driving the reaction to completion, Truppo et al. (2009) developed an elegant multi-enzymatic system for the HTP-screening and scale-up of TA catalyzed reactions (see Figure 3b). In this system, pyruvate which is generated through the TA reaction is reduced to L-lactate by a lactate dehydrogenase (LDH). Recycling of the LDH cofactor NADH by glucose dehydrogenase (GDH) ultimately leads to the formation of gluconic acid and thus to a pH drop. The progression of the reaction can be measured by monitoring the change of absorbance of a pH indicator (phenol red). This system proved to be especially useful for rapid scale-up. While the system was only tested with ketones as substrate, theoretically β-keto acids could also be employed. Limits of the reaction are that only pyruvate dependent TAs can be tested, no information on the enantioselectivity or enantiopreference of the studied enzyme can be gained and that as it is a multi-enzymatic system, reaction conditions can only be altered marginally. Additionally, most β-keto acids are instable due to spontaneous decarboxylation. A potential solution to this crucial problem is discussed in the conclusion section. A simple assay for the screening of ω-TAs has been developed by Schätzle et al. (2009). The assay is based on the transamination of the model substrate α-methylbenzylamine to acetophenone (see Figure 3c). Acetophenone exhibits high absorbance around 245 nm. While this assay does not directly screen for activity towards β-amino acids, α-methylbenzylamine constitutes a good model substrate for ω-TAs which also possess activity towards short-chain aliphatic β-amino acids. Advantages of this assay are its applicability to cell extracts, the possibility to determine the enantiopreference by using enantiopure α-methylbenzylamine and its high sensitivity. The same group recently published another assay which allows the determination of amino donor specificity (see Figure 3d) (Schätzle et al. 2010). The principle of the assay differs from all other presented methods as it measures the change of conductivity of the reaction mixture. The progression of the reaction results in a decrease of conductivity as charged compounds (a positively charged amine and a negatively charged keto acid) are converted to non-conducting products (a non-charged ketone and a zwitterionic amino acid). Advantages of this assay are the broad spectra of amino donors and amino acceptors which can be used and its high sensitivity. Yet the mode of analysis itself makes the simultaneous measurement of many samples cumbersome as each reaction tube or well would have to be equipped with an electrode. This conductivity assay should also work using a β-keto acid instead of an α-keto acid, leading to the zwitterionic β-amino acid and an uncharged ketone. However, the potential spontaneous decarboxylation of the charged β-keto acid to an uncharged compound might lead to some conductivity decrease even without TA activity. Hopwood et al. (2011) recently described a multi-enzymatic reaction system employing an amino acid oxidase which converts the co-product of the transamination reaction, D- or L-alanine, to the corresponding imine (see Figure 3e). The hereby produced H_2_O_2 _oxidizes, catalyzed by horse radish peroxidase, the dye pyrogallol red. The reaction can be monitored by measuring the decrease in absorbance around 540 nm. This method allows the use of many different amine donors as well as the determination of the enantiopreference of the transaminase. When using a β-amino acid instead of an amine, false positive results might occur if the amino acid oxidase (AAO) also oxidizes the educt and not only the coproduct. However, to our knowledge such AAO activity towards β-amino acids has never been reported. Further drawbacks include that free amino acids distort the results, which makes enzyme purification necessary and that as it is a multi-enzymatic reaction system, reaction conditions can only be changed marginally.

## Conclusions and future areas of research

TAs possess a great potential for the enzymatic synthesis of enantiopure β-amino acids as these enzymes offer the possibility to gain a 100% yield in contrast to the conventional kinetic resolutions using other biocatalysts (see introduction). Transaminases are commonly used tools in the synthesis of various chemicals and pharmaceuticals. Thus production and purification of these enzymes in bulk quantities is well-established, and so is immobilization. Additionally, several process parameters for biotechnological applications are well investigated for both kinetic resolutions and asymmetric syntheses, e.g. the usage of different (co-)solvents and variation of pH and PLP concentration as well as different strategies of product removal (Koszelewski et al. 2008). Of special importance are the thoroughly tried and tested methods to shift the equilibrium to the product side by removal of the coproduct (kinetic resolutions) or degradation of the coproduct/recycling of the amino donor by different enzymes in asymmetric synthesis (Koszelewski et al. 2010).

All these benefits for technical applications are not established with aminomutases and only in part with hydantoinases (as described above), so TAs appear as the most promising candidates among the potential biocatalysts for a 100% yield synthesis of β-amino acids.

A key step in fulfilling this potential is the discovery of new TAs with a broader substrate spectrum and different enantioselectivity. This will be greatly facilitated by the HTP-activity assays described in this article, which allow for time and cost efficient screening, characterization and enzyme optimization. As has been discussed, transaminases which act on aliphatic β-amino acids are abundant, while only a small number of transaminases which act on aromatic β-amino acids have been described. The application of protein engineering to enhance the activity of TAs towards aromatic β-amino acids has been only moderately successful. The crystal structure of the transaminase of *Mesorhizobium *sp. LUK will lead to a more targeted approach in protein engineering.

Little attention so far has been paid to the preparation of the thermodynamically instable β-keto substrate. A possible route is the enzymatic hydrolysis of the corresponding β-keto-ester. Although such a route has already been described ([Bibr B3][Bibr B33]) further optimization will be necessary for the development of efficient processes as the reported yields are quite low (~20%). This might be due to the loss of the intermediary β-keto acid via decarboxylation to acetophenone (which is not discussed in Kim et al. 2007).

This instability of the potential substrates indeed appears as the bottleneck in β-amino acid synthesis via transaminases. A more elegant solution could be obtained by changing the sequence in this coupled enzyme reaction: If the TA accepts the stable β-keto ester (1) the intermediary synthesis of the β-keto acid can be circumvented and (2) the adjacent application of an enantioselective lipase to subsequently cleave the intermediary chiral β-amino acid ester would further enhance the ee value. As the authors assume this can not happen in the reported reaction due to a mandatory anchoring of the substrate's carboxylic group by one of the two binding pockets around the PLP-lysine Schiff base (the external aldimine intermediate) which is certainly not possible with the esterified substrate (Kim et al. 2007).

However, advances in protein design and substrate modeling might help to overcome this problem, as for example the excavation of the small pocket of the commonly used transaminase ATA-117 allowed the *(R)*-selective amination of prositagliptin ketone to the antidiabetic compound sitagliptin (Savile et al. 2010) which was not successful before using a variety of unmodified TAs due to sterical hindrance.

Not least, the potential of TAs for the asymmetric synthesis of β-amino acids can be assumed to be even much higher, as most of the numerous commercially available TAs have never been tested with β-amino acids or β-keto acids as substrates (the latter due to their instability mentioned above). So the conclusive key step will be a modification of the HTP-assays described to allow a fast and comprehensive screening for β-TA activity.

## Competing interests

The authors declare that they have no competing interests.
